# (*meso*-5,7,7,12,14,14-Hexamethyl-1,4,8,11-tetra­azacyclo­tetra­deca-4,11-diene)copper(II) bis­[*O*,*O*′-bis­(4-methyl­phen­yl) dithio­phosphate]

**DOI:** 10.1107/S1600536810009815

**Published:** 2010-03-20

**Authors:** Lin-Xin He, Li-Ke Zou, Bin Xie, Yang-Guang Xiang, Jian-Shen Feng

**Affiliations:** a College of Chemistry and Pharmaceutical Engineering, Sichuan University of Science and Engineering , 643000 Zigong, Sichuan, People’s Republic of China

## Abstract

The title compound, [Cu(C_16_H_32_N_4_)](C_14_H_14_O_2_PS_2_)_2_ or [Cu(*trans*[14]dien)][S_2_P(OC_6_H_4_Me-4)_2_]_2_, where *trans*[14]dien is *meso*-5,7,7,12,14,14-hexa­methyl-1,4,8,11-tetra­azacyclo­tetra­deca-4,11-diene, was obtained by the reaction of [Cu(*trans*[14]dien)](ClO_4_)_2_ and [(C_2_H_5_)_2_NH]_2_ [S_2_P(OC_6_H_4_Me-4)_2_]_2_. The Cu^II^ atom lies on a centre of inversion and possesses a relatively undistorted square-planar coordination arrangement with four N atoms of the macrocyclic tetra­mine *trans*[14]dien [Cu—N = 1.9716 (19) and 2.0075 (19) Å]. The two uncoordinated [(4-MeC_6_H_4_O)_2_PS_2_]^−^ groups act as counter-ions to balance the charge and inter­act with the [Cu(*trans*[14]dien)]^2+ ^complex cation through N—H⋯S hydrogen bonds.

## Related literature

For general background to the potential uses of copper(I) and copper(II) complexes with *O*,*O*′-dialkyl­dithio­phosphate ligands, see: Drew *et al.* (1987 ▶); Liu *et al.* (1995 ▶); Liaw *et al.* (2005 ▶). For a related structure, see: Xie *et al.* (2009 ▶). For bond-length data, see: Allen *et al.* (1987 ▶).
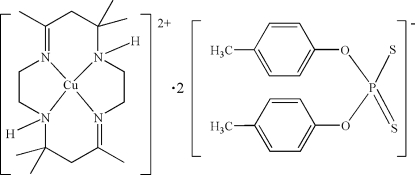


## Experimental

### 

#### Crystal data

[Cu(C_16_H_32_N_4_)](C_14_H_14_O_2_PS_2_)_2_
*M* *_r_* = 962.68
Triclinic, 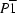

*a* = 8.1043 (9) Å
*b* = 10.2120 (11) Å
*c* = 15.8435 (17) Å
α = 82.456 (2)°
β = 79.623 (2)°
γ = 70.797 (2)°
*V* = 1214.3 (2) Å^3^
*Z* = 1
Mo *K*α radiation
μ = 0.73 mm^−1^
*T* = 273 K
0.18 × 0.13 × 0.08 mm

#### Data collection

Bruker SMART CCD area-detector diffractometer
Absorption correction: multi-scan (*SADABS*; Bruker, 2001 ▶) *T*_min_ = 0.862, *T*_max_ = 0.924
6424 measured reflections
4266 independent reflections
3527 reflections with *I* > 2σ(*I*)
*R*_int_ = 0.015

#### Refinement

*R*[*F* ^2^ > 2σ(*F* ^2^)] = 0.034
*wR*(*F* ^2^) = 0.092
*S* = 1.03
4266 reflections
273 parameters
H-atom parameters constrained
Δρ_max_ = 0.27 e Å^−3^
Δρ_min_ = −0.23 e Å^−3^

### 

Data collection: *SMART* (Bruker, 2001 ▶); cell refinement: *SAINT* (Bruker, 2001 ▶); data reduction: *SAINT*; program(s) used to solve structure: *SHELXTL* (Sheldrick, 2008 ▶); program(s) used to refine structure: *SHELXTL*; molecular graphics: *ORTEP-3 for Windows* (Farrugia,1997 ▶); software used to prepare material for publication: *SHELXTL*.

## Supplementary Material

Crystal structure: contains datablocks I, global. DOI: 10.1107/S1600536810009815/dn2547sup1.cif

Structure factors: contains datablocks I. DOI: 10.1107/S1600536810009815/dn2547Isup2.hkl

Additional supplementary materials:  crystallographic information; 3D view; checkCIF report

## Figures and Tables

**Table 1:** Hydrogen-bond geometry (Å, °)

*D*—H⋯*A*	*D*—H	H⋯*A*	*D*⋯*A*	*D*—H⋯*A*
N2—H1⋯S1^i^	0.86	2.77	3.559 (2)	153
N2—H1⋯S2^i^	0.86	2.83	3.477 (2)	134

Symmetry code: (i) 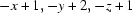.

**Fig. 1.**
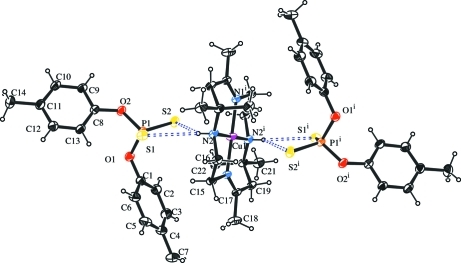
The molecular structure of the title compound, showing the atom-numbering scheme. Displacement ellipsoids are drawn at the 30% probability level. H atoms are represented as small spheres of arbitary radii. Hydrogen-bonds are shown as dashed lines.[Symmetry code: (i) -x + 1, -y + 2, -z + 1].

## supplementary crystallographic information

### Comment

The complexes of copper(I) and copper(II) with *O*,*O*' -dialkyldithiophosphate ligands (DDP), have been explored extensively in the past decades because of their potential use as anti-oxidants, additives to lubricating oils, flotation reagents, insecticides (Drew *et al.*, 1987;Liu *et al.*, 1995; Liaw *et al.*, 2005). The reactions between copper(II) and DDP rarely give stable copper(II) complexes, because the DDP ligands act as a reducing agent to form copper(I) complexs. However, the copper(II) can be stabilized by the formation of adducts with tetradentate nitrogen-donor ligands, e.g. macrocyclic tetramine, when reacting with DDP. We report here the structure of a copper(II) adducts, ([Cu(*trans*[14]dien)][S_2_P(OC_6_H_4_Me-4)_2_]_2_, where *trans*[14]dien is *meso*-5,7,7,12,14,14- hexamethyl-1,4,8,11-tetraazacyclotetradeca-4,11-diene.

In the complex cation [Cu(*trans*[14]dien)]^2+^, the Cu^II^ atom which lies on an inversion centre, is coordinated by four N atoms of the macrocyclic tetramine *trans*[14]dien exhibiting a relatively undistorted square-planar geometry (Fig.1). The two uncoordinated *O*,*O*'-di(4-methylphenyl) dithiophosphates only act as counter-ions to balance the charge and interact with the complex cation through N—H···S hydrogen bonds (Table 1). Similar structure is seen in the analogous adduct, ([Ni(*trans*[14]dien)] [S_2_P(OC_6_H_4_Me-4)_2_]_2_ (Xie *et al.*, 2009). All the bond lengths and angles in the complex are generally within normal ranges (Allen *et al.*, 1987).

### Experimental

*meso*-5,7,7,12,14,14-Hexamethyl-1,4,8,11-tetraazacyclotetradeca-4, 11-diene nickel(II) diperchlorate(1 mmol, 0.543 g) was added to a solution of diethylammonium *O*,*O*'-di(4-methylphenyl)dithiophosphate (2 mmol, 0.767 g) in 60 ml methanol. The mixture was refluxed for 6 h at 70°C and then filtered. The filtrate was kept at room temperature and purple block crystals suitable for X-ray diffraction studies were obtained after one week.

### Refinement

H atoms on C were fixed geometrically and treated as riding, with C—H = 0.97 Å (methylene), 0.96Å (methyl) or 0.93Å (aromatic) and U_iso_(H) = 1.2U_eq_(*C*,methylene and aromatic) or U_iso_(H) = 1.5U_eq_(*C*,methyl). The H atoms on N were determined with difference Fourier syntheses and refined isotropically.

### Crystal data

**Table:**

[Cu(C_16_H_32_N_4_)](C_14_H_14_O_2_PS_2_)_2_	*Z* = 1
*M**_r_* = 962.68	*F*(000) = 507
Triclinic, *P*1	*D*_x_ = 1.316 Mg m^−^^3^
Hall symbol: -P 1	Mo *K*α radiation, λ = 0.71073 Å
*a* = 8.1043 (9) Å	Cell parameters from 2875 reflections
*b* = 10.2120 (11) Å	θ = 2.6–26.6°
*c* = 15.8435 (17) Å	µ = 0.73 mm^−^^1^
α = 82.456 (2)°	*T* = 273 K
β = 79.623 (2)°	Block, purple
γ = 70.797 (2)°	0.18 × 0.13 × 0.08 mm
*V* = 1214.3 (2) Å^3^	

### Data collection

**Table:**

Bruker SMART CCD area-detector diffractometer	4266 independent reflections
Radiation source: fine-focus sealed tube	3527 reflections with *I* > 2σ(*I*)
graphite	*R*_int_ = 0.015
phi and ω scans	θ_max_ = 25.0°, θ_min_ = 1.3°
Absorption correction: multi-scan (*SADABS*; Bruker, 2001)	*h* = −9→9
*T*_min_ = 0.862, *T*_max_ = 0.924	*k* = −12→12
6424 measured reflections	*l* = −14→18

### Refinement

**Table:**

Refinement on *F*^2^	Primary atom site location: structure-invariant direct methods
Least-squares matrix: full	Secondary atom site location: difference Fourier map
*R*[*F*^2^ > 2σ(*F*^2^)] = 0.034	Hydrogen site location: inferred from neighbouring sites
*wR*(*F*^2^) = 0.092	H-atom parameters constrained
*S* = 1.03	*w* = 1/[σ^2^(*F*_o_^2^) + (0.042*P*)^2^ + 0.4609*P*] where *P* = (*F*_o_^2^ + 2*F*_c_^2^)/3
4266 reflections	(Δ/σ)_max_ = 0.002
273 parameters	Δρ_max_ = 0.27 e Å^−^^3^
0 restraints	Δρ_min_ = −0.23 e Å^−^^3^

### Special details

**Table:**

Geometry. All esds (except the esd in the dihedral angle between two l.s. planes) are estimated using the full covariance matrix. The cell esds are taken into account individually in the estimation of esds in distances, angles and torsion angles; correlations between esds in cell parameters are only used when they are defined by crystal symmetry. An approximate (isotropic) treatment of cell esds is used for estimating esds involving l.s. planes.
Refinement. Refinement of *F*^2^ against ALL reflections. The weighted *R*-factor wR and goodness of fit *S* are based on *F*^2^, conventional *R*-factors *R* are based on *F*, with *F* set to zero for negative *F*^2^. The threshold expression of *F*^2^ > σ(*F*^2^) is used only for calculating *R*-factors(gt) etc. and is not relevant to the choice of reflections for refinement. *R*-factors based on *F*^2^ are statistically about twice as large as those based on *F*, and *R*- factors based on ALL data will be even larger.

### Fractional atomic coordinates and isotropic or equivalent isotropic displacement parameters (Å^2^)

**Table:**

	*x*	*y*	*z*	*U*_iso_*/*U*_eq_	
Cu1	0.5000	1.0000	0.5000	0.04864 (14)	
N1	0.3721 (2)	0.97265 (18)	0.41247 (12)	0.0433 (4)	
N2	0.3604 (2)	1.20195 (18)	0.48345 (13)	0.0455 (5)	
H1	0.4288	1.2261	0.4408	0.055*	
C15	0.2619 (3)	1.1075 (2)	0.37755 (16)	0.0504 (6)	
H15A	0.1598	1.0966	0.3589	0.060*	
H15B	0.3291	1.1435	0.3285	0.060*	
C16	0.2037 (3)	1.2058 (2)	0.44768 (17)	0.0537 (6)	
H16A	0.1464	1.2994	0.4246	0.064*	
H16B	0.1202	1.1781	0.4924	0.064*	
C17	0.3685 (3)	0.8572 (2)	0.39127 (15)	0.0457 (5)	
C18	0.2533 (4)	0.8444 (3)	0.3313 (2)	0.0770 (9)	
H18A	0.1657	0.9327	0.3219	0.116*	
H18B	0.1961	0.7766	0.3559	0.116*	
H18C	0.3240	0.8157	0.2774	0.116*	
C19	0.4815 (3)	0.7214 (2)	0.42779 (17)	0.0520 (6)	
H19A	0.4940	0.6512	0.3893	0.062*	
H19B	0.4172	0.6973	0.4822	0.062*	
C20	0.6663 (3)	0.7096 (2)	0.44375 (16)	0.0479 (6)	
C21	0.7585 (4)	0.5565 (2)	0.4696 (2)	0.0673 (8)	
H21A	0.8772	0.5448	0.4778	0.101*	
H21B	0.7610	0.5004	0.4250	0.101*	
H21C	0.6952	0.5282	0.5222	0.101*	
C22	0.7720 (3)	0.7578 (3)	0.36425 (18)	0.0627 (7)	
H22A	0.7205	0.8560	0.3518	0.094*	
H22B	0.7712	0.7095	0.3163	0.094*	
H22C	0.8915	0.7384	0.3741	0.094*	
S1	0.49170 (8)	0.58786 (7)	0.69163 (5)	0.05677 (18)	
S2	0.20902 (8)	0.88776 (7)	0.62430 (5)	0.05820 (19)	
P1	0.26451 (8)	0.73303 (6)	0.71235 (4)	0.04638 (17)	
O1	0.2353 (2)	0.78874 (18)	0.80671 (11)	0.0551 (4)	
O2	0.0998 (2)	0.67359 (18)	0.72844 (12)	0.0593 (5)	
C1	0.3506 (3)	0.8508 (3)	0.82839 (15)	0.0527 (6)	
C2	0.3047 (4)	0.9926 (3)	0.82335 (18)	0.0678 (7)	
H2	0.1998	1.0474	0.8040	0.081*	
C3	0.4174 (6)	1.0532 (4)	0.8476 (2)	0.0854 (10)	
H3	0.3861	1.1497	0.8445	0.102*	
C4	0.5727 (5)	0.9756 (4)	0.8758 (2)	0.0829 (10)	
C5	0.6147 (5)	0.8334 (4)	0.8803 (2)	0.0824 (9)	
H5	0.7200	0.7784	0.8991	0.099*	
C6	0.5046 (4)	0.7706 (3)	0.85759 (18)	0.0679 (7)	
H6	0.5344	0.6741	0.8620	0.082*	
C7	0.6929 (7)	1.0447 (5)	0.9027 (3)	0.1315 (18)	
H7A	0.6468	1.1437	0.8911	0.197*	
H7B	0.8091	1.0110	0.8709	0.197*	
H7C	0.6985	1.0232	0.9632	0.197*	
C8	0.0709 (3)	0.5670 (2)	0.78801 (16)	0.0500 (6)	
C9	−0.0702 (3)	0.5272 (3)	0.78031 (17)	0.0571 (6)	
H9	−0.1370	0.5682	0.7363	0.069*	
C10	−0.1141 (4)	0.4258 (3)	0.83797 (19)	0.0654 (7)	
H10	−0.2101	0.3989	0.8319	0.078*	
C11	−0.0189 (4)	0.3637 (3)	0.90417 (19)	0.0637 (7)	
C12	0.1238 (4)	0.4049 (3)	0.9097 (2)	0.0735 (8)	
H12	0.1912	0.3636	0.9535	0.088*	
C13	0.1709 (4)	0.5060 (3)	0.85227 (19)	0.0664 (8)	
H13	0.2683	0.5319	0.8573	0.080*	
C14	−0.0684 (5)	0.2542 (4)	0.9688 (2)	0.0930 (11)	
H14A	−0.0184	0.1649	0.9456	0.140*	
H14B	−0.1946	0.2769	0.9803	0.140*	
H14C	−0.0234	0.2515	1.0212	0.140*	

### Atomic displacement parameters (Å^2^)

**Table:**

	*U*^11^	*U*^22^	*U*^33^	*U*^12^	*U*^13^	*U*^23^
Cu1	0.0435 (2)	0.0334 (2)	0.0719 (3)	−0.00251 (17)	−0.0267 (2)	−0.01418 (18)
N1	0.0372 (10)	0.0391 (10)	0.0572 (12)	−0.0120 (8)	−0.0136 (8)	−0.0074 (9)
N2	0.0381 (10)	0.0374 (10)	0.0615 (12)	−0.0095 (8)	−0.0103 (9)	−0.0079 (9)
C15	0.0434 (13)	0.0466 (13)	0.0644 (16)	−0.0120 (11)	−0.0225 (11)	−0.0016 (11)
C16	0.0428 (13)	0.0430 (13)	0.0735 (17)	−0.0054 (11)	−0.0197 (12)	−0.0040 (12)
C17	0.0364 (12)	0.0483 (13)	0.0563 (14)	−0.0154 (10)	−0.0050 (10)	−0.0147 (11)
C18	0.0620 (17)	0.076 (2)	0.104 (2)	−0.0157 (15)	−0.0330 (17)	−0.0351 (18)
C19	0.0513 (14)	0.0393 (13)	0.0718 (16)	−0.0188 (11)	−0.0079 (12)	−0.0164 (11)
C20	0.0424 (12)	0.0353 (12)	0.0661 (15)	−0.0086 (10)	−0.0088 (11)	−0.0127 (11)
C21	0.0658 (17)	0.0361 (13)	0.096 (2)	−0.0034 (12)	−0.0184 (15)	−0.0155 (13)
C22	0.0513 (15)	0.0569 (16)	0.0782 (19)	−0.0167 (13)	0.0024 (13)	−0.0166 (14)
S1	0.0490 (4)	0.0516 (4)	0.0687 (4)	−0.0070 (3)	−0.0151 (3)	−0.0149 (3)
S2	0.0438 (3)	0.0557 (4)	0.0707 (4)	−0.0152 (3)	−0.0071 (3)	0.0078 (3)
P1	0.0383 (3)	0.0466 (3)	0.0570 (4)	−0.0161 (3)	−0.0082 (3)	−0.0049 (3)
O1	0.0499 (10)	0.0603 (10)	0.0573 (10)	−0.0226 (8)	0.0021 (8)	−0.0119 (8)
O2	0.0500 (10)	0.0637 (11)	0.0735 (12)	−0.0307 (9)	−0.0224 (9)	0.0144 (9)
C1	0.0565 (15)	0.0581 (15)	0.0452 (14)	−0.0204 (12)	0.0002 (11)	−0.0142 (11)
C2	0.080 (2)	0.0573 (17)	0.0662 (18)	−0.0202 (15)	−0.0092 (15)	−0.0124 (14)
C3	0.130 (3)	0.066 (2)	0.075 (2)	−0.047 (2)	−0.012 (2)	−0.0162 (16)
C4	0.110 (3)	0.101 (3)	0.0615 (19)	−0.057 (2)	−0.0210 (18)	−0.0138 (17)
C5	0.087 (2)	0.102 (3)	0.069 (2)	−0.031 (2)	−0.0292 (17)	−0.0147 (18)
C6	0.0755 (19)	0.0644 (17)	0.0664 (18)	−0.0162 (15)	−0.0215 (15)	−0.0134 (14)
C7	0.183 (5)	0.171 (4)	0.100 (3)	−0.118 (4)	−0.049 (3)	−0.016 (3)
C8	0.0468 (13)	0.0470 (13)	0.0595 (15)	−0.0190 (11)	−0.0076 (11)	−0.0040 (11)
C9	0.0477 (14)	0.0660 (17)	0.0648 (16)	−0.0268 (13)	−0.0121 (12)	−0.0016 (13)
C10	0.0578 (16)	0.0706 (18)	0.079 (2)	−0.0379 (14)	−0.0041 (14)	−0.0082 (15)
C11	0.0751 (19)	0.0575 (16)	0.0657 (18)	−0.0340 (15)	−0.0027 (15)	−0.0052 (13)
C12	0.088 (2)	0.0710 (19)	0.0742 (19)	−0.0385 (17)	−0.0310 (17)	0.0119 (15)
C13	0.0683 (17)	0.0664 (17)	0.0800 (19)	−0.0395 (15)	−0.0285 (15)	0.0128 (15)
C14	0.122 (3)	0.087 (2)	0.084 (2)	−0.061 (2)	−0.006 (2)	0.0100 (19)

### Geometric parameters (Å, °)

**Table:**

Cu1—N1^i^	1.9714 (18)	P1—O2	1.6075 (16)
Cu1—N1	1.9714 (18)	P1—O1	1.6193 (18)
Cu1—N2^i^	2.0079 (18)	O1—C1	1.398 (3)
Cu1—N2	2.0080 (17)	O2—C8	1.396 (3)
N1—C17	1.278 (3)	C1—C2	1.367 (4)
N1—C15	1.473 (3)	C1—C6	1.371 (4)
N2—C16	1.468 (3)	C2—C3	1.388 (4)
N2—C20^i^	1.499 (3)	C2—H2	0.9300
N2—H1	0.8576	C3—C4	1.366 (5)
C15—C16	1.502 (3)	C3—H3	0.9300
C15—H15A	0.9700	C4—C5	1.373 (5)
C15—H15B	0.9700	C4—C7	1.519 (4)
C16—H16A	0.9700	C5—C6	1.376 (4)
C16—H16B	0.9700	C5—H5	0.9300
C17—C18	1.491 (3)	C6—H6	0.9300
C17—C19	1.497 (3)	C7—H7A	0.9600
C18—H18A	0.9600	C7—H7B	0.9600
C18—H18B	0.9600	C7—H7C	0.9600
C18—H18C	0.9600	C8—C9	1.363 (3)
C19—C20	1.527 (3)	C8—C13	1.371 (4)
C19—H19A	0.9700	C9—C10	1.382 (4)
C19—H19B	0.9700	C9—H9	0.9300
C20—N2^i^	1.499 (3)	C10—C11	1.376 (4)
C20—C22	1.513 (4)	C10—H10	0.9300
C20—C21	1.531 (3)	C11—C12	1.375 (4)
C21—H21A	0.9600	C11—C14	1.518 (4)
C21—H21B	0.9600	C12—C13	1.389 (4)
C21—H21C	0.9600	C12—H12	0.9300
C22—H22A	0.9600	C13—H13	0.9300
C22—H22B	0.9600	C14—H14A	0.9600
C22—H22C	0.9600	C14—H14B	0.9600
S1—P1	1.9519 (9)	C14—H14C	0.9600
S2—P1	1.9556 (9)		
			
N1^i^—Cu1—N1	180.000 (1)	H22A—C22—H22C	109.5
N1^i^—Cu1—N2^i^	85.26 (7)	H22B—C22—H22C	109.5
N1—Cu1—N2^i^	94.74 (7)	O2—P1—O1	97.09 (9)
N1^i^—Cu1—N2	94.74 (7)	O2—P1—S1	112.84 (7)
N1—Cu1—N2	85.26 (7)	O1—P1—S1	110.67 (7)
N2^i^—Cu1—N2	180.00 (12)	O2—P1—S2	105.49 (7)
C17—N1—C15	121.97 (19)	O1—P1—S2	111.15 (7)
C17—N1—Cu1	127.29 (16)	S1—P1—S2	117.61 (4)
C15—N1—Cu1	110.51 (13)	C1—O1—P1	120.99 (14)
C16—N2—C20^i^	117.81 (18)	C8—O2—P1	128.25 (15)
C16—N2—Cu1	105.96 (13)	C2—C1—C6	120.3 (3)
C20^i^—N2—Cu1	117.25 (14)	C2—C1—O1	119.2 (2)
C16—N2—H1	105.2	C6—C1—O1	120.4 (2)
C20^i^—N2—H1	109.6	C1—C2—C3	118.8 (3)
Cu1—N2—H1	98.8	C1—C2—H2	120.6
N1—C15—C16	107.80 (19)	C3—C2—H2	120.6
N1—C15—H15A	110.1	C4—C3—C2	122.1 (3)
C16—C15—H15A	110.1	C4—C3—H3	119.0
N1—C15—H15B	110.1	C2—C3—H3	119.0
C16—C15—H15B	110.1	C3—C4—C5	117.7 (3)
H15A—C15—H15B	108.5	C3—C4—C7	121.0 (4)
N2—C16—C15	108.29 (19)	C5—C4—C7	121.3 (4)
N2—C16—H16A	110.0	C4—C5—C6	121.5 (3)
C15—C16—H16A	110.0	C4—C5—H5	119.3
N2—C16—H16B	110.0	C6—C5—H5	119.3
C15—C16—H16B	110.0	C1—C6—C5	119.7 (3)
H16A—C16—H16B	108.4	C1—C6—H6	120.2
N1—C17—C18	124.4 (2)	C5—C6—H6	120.2
N1—C17—C19	121.0 (2)	C4—C7—H7A	109.5
C18—C17—C19	114.6 (2)	C4—C7—H7B	109.5
C17—C18—H18A	109.5	H7A—C7—H7B	109.5
C17—C18—H18B	109.5	C4—C7—H7C	109.5
H18A—C18—H18B	109.5	H7A—C7—H7C	109.5
C17—C18—H18C	109.5	H7B—C7—H7C	109.5
H18A—C18—H18C	109.5	C9—C8—C13	120.3 (2)
H18B—C18—H18C	109.5	C9—C8—O2	115.3 (2)
C17—C19—C20	119.01 (19)	C13—C8—O2	124.3 (2)
C17—C19—H19A	107.6	C8—C9—C10	120.0 (3)
C20—C19—H19A	107.6	C8—C9—H9	120.0
C17—C19—H19B	107.6	C10—C9—H9	120.0
C20—C19—H19B	107.6	C11—C10—C9	121.5 (3)
H19A—C19—H19B	107.0	C11—C10—H10	119.3
N2^i^—C20—C22	111.23 (19)	C9—C10—H10	119.3
N2^i^—C20—C19	105.85 (18)	C12—C11—C10	117.2 (3)
C22—C20—C19	111.6 (2)	C12—C11—C14	121.0 (3)
N2^i^—C20—C21	110.8 (2)	C10—C11—C14	121.8 (3)
C22—C20—C21	109.8 (2)	C11—C12—C13	122.3 (3)
C19—C20—C21	107.38 (19)	C11—C12—H12	118.9
C20—C21—H21A	109.5	C13—C12—H12	118.9
C20—C21—H21B	109.5	C8—C13—C12	118.7 (3)
H21A—C21—H21B	109.5	C8—C13—H13	120.6
C20—C21—H21C	109.5	C12—C13—H13	120.6
H21A—C21—H21C	109.5	C11—C14—H14A	109.5
H21B—C21—H21C	109.5	C11—C14—H14B	109.5
C20—C22—H22A	109.5	H14A—C14—H14B	109.5
C20—C22—H22B	109.5	C11—C14—H14C	109.5
H22A—C22—H22B	109.5	H14A—C14—H14C	109.5
C20—C22—H22C	109.5	H14B—C14—H14C	109.5
			
C17—N1—C15—C16	143.9 (2)	C1—C2—C3—C4	0.3 (5)
C20^i^—N2—C16—C15	−178.75 (19)	C2—C3—C4—C5	−0.5 (5)
N1—C15—C16—N2	50.8 (3)	C2—C3—C4—C7	−179.5 (3)
C15—N1—C17—C18	−1.0 (4)	C3—C4—C5—C6	−0.2 (5)
C15—N1—C17—C19	−179.9 (2)	C7—C4—C5—C6	178.7 (3)
N1—C17—C19—C20	−36.3 (3)	C2—C1—C6—C5	−1.2 (4)
C18—C17—C19—C20	144.7 (2)	O1—C1—C6—C5	−178.7 (3)
C17—C19—C20—N2^i^	68.8 (3)	C4—C5—C6—C1	1.1 (5)
C17—C19—C20—C22	−52.3 (3)	P1—O2—C8—C9	−172.40 (19)
C17—C19—C20—C21	−172.8 (2)	P1—O2—C8—C13	9.3 (4)
O2—P1—O1—C1	178.84 (18)	C13—C8—C9—C10	0.6 (4)
S1—P1—O1—C1	61.14 (19)	O2—C8—C9—C10	−177.8 (2)
S2—P1—O1—C1	−71.48 (18)	C8—C9—C10—C11	0.5 (4)
O1—P1—O2—C8	−62.5 (2)	C9—C10—C11—C12	−1.2 (4)
S1—P1—O2—C8	53.5 (2)	C9—C10—C11—C14	178.8 (3)
S2—P1—O2—C8	−176.78 (19)	C10—C11—C12—C13	0.8 (5)
P1—O1—C1—C2	98.2 (2)	C14—C11—C12—C13	−179.2 (3)
P1—O1—C1—C6	−84.4 (3)	C9—C8—C13—C12	−1.0 (4)
C6—C1—C2—C3	0.5 (4)	O2—C8—C13—C12	177.3 (3)
O1—C1—C2—C3	178.0 (2)	C11—C12—C13—C8	0.2 (5)

Symmetry codes: (i) −*x*+1, −*y*+2, −*z*+1.

### Hydrogen-bond geometry (Å, °)

**Table:**

*D*—H···*A*	*D*—H	H···*A*	*D*···*A*	*D*—H···*A*
N2—H1···S1^i^	0.86	2.77	3.559 (2)	153
N2—H1···S2^i^	0.86	2.83	3.477 (2)	134

Symmetry codes: (i) −*x*+1, −*y*+2, −*z*+1.
